# AMAFRICA, a patient-navigator program for accompanying lymphoma patients during chemotherapy in Ivory Coast: a prospective randomized study

**DOI:** 10.1186/s12885-019-6478-3

**Published:** 2019-12-23

**Authors:** K. G. Koffi, D. A. Silué, C. Laurent, K. Boidy, S. Koui, G. Compaci, Z. H. Adeba, I. Kamara, R. P. Botty, A. S. Bognini, I. Sanogo, F. Despas, G. Laurent

**Affiliations:** 1 Hematology Teaching Hospital of Yopougon University Medical Center, Abidjan, Côte d’Ivoire; 2 Hematology Department of Toulouse University Medical Center, Toulouse, France

**Keywords:** Malignant lymphoma, AMAFRICA program

## Abstract

**Background:**

Previous studies have indicated that accompanying socially underserved cancer patients through Patient Navigator (PN) or PN-derived procedures improves therapy management and reassurance. At the Cancer Institute of Toulouse-Oncopole (France), we have implemented AMA (Ambulatory Medical Assistance), a PN-based procedure adapted for malignant lymphoma (ML) patients under therapy. We found that AMA improves adherence to chemotherapy and safety. In low-middle income countries (LMIC), refusal and abandonment were documented as major adverse factors for cancer therapy. We reasoned that AMA could improve clinical management of ML patients in LMIC.

**Methods:**

This study was set up in the Abidjan University Medical Center (Ivory Coast) in collaboration with Toulouse. One hundred African patients were randomly assigned to either an AMA or control group. Main criteria of judgment were refusal and abandonment of CHOP or ABVD chemotherapy.

**Results:**

We found that AMA was feasible and had significant impact on refusal and abandonment. However, only one third of patients completed their therapy in both groups. No differences were noted in terms of complete response rate (CR) (16% based on intent-to-treat) and median overall survival (OS) (6 months). The main reason for refusal and abandonment was limitation of financial resources.

**Conclusion:**

Altogether, this study showed that PN may reduce refusal and abandonment of treatment. However, due to insufficient health care coverage, its ultimate impact on OS remains limited.

## Background

The treatment of malignant lymphoma (ML) in low-middle income countries (LMICs) faces many obstacles. Indeed, delayed diagnosis, refusal or abandonment of therapy, suboptimal treatment adherence, and inadequate supportive therapy, contribute among other parameters to poor outcome.

These obstacles are themselves related to adverse factors such as limitation of universal health care, private or familial financial constraints, low number of specialists, limited access to imaging (e.g. CT scan) and to routine laboratory practices, including biopsy specimen analysis. Transportation difficulties or interference with traditional medicine may also occasionally play a role.

Historically, the Patient Navigator (PN) was promoted in the late 80s by Dr. Freeman in New York City, to help underprivileged patients suffering from breast cancers at the earliest phase of their health care trajectory [1]. From the start, the PN has been proposed as an accompanying procedure based on coordinated interactions between the oncologist and the patient through phone calls at the patient’s home or visits by volunteers or nurses during the initial phase of the patient’s trajectory. PN was found to shorten the delay between screening and initiation of therapy as well as increase patient reassurance [1]. In the US, PN was found to be efficient for reducing health care disparities and it is now supported by the Patient Protection and Affordability Act [2]. Surprisingly, PN received little attention in LMIC with some exceptions, such as in Brazil [3].

PN has been applied through different variants, among which the AMA (Ambulatory Medical Assistance) procedure implemented at the Hematology Department of Toulouse University Medical Center (France) for lymphoma patients treated with chemotherapy [4]. AMA consists of a systematic weekly phone call to the patient’s home by a specialized nurse (nurse navigator/NN) who collects all information concerning drug-induced toxicities. Based on an algorithm, the signs are considered as insignificant, manageable by the nurse, or requiring immediate intervention of the oncologist. AMA was found to be feasible and highly efficient for detecting and managing complications during chemotherapy. AMA benefited from overwhelming support from patients and caregivers. AMA also appeared to be efficient in its triage function, with considerable time saving for medical staff. More recently, a randomized study demonstrated that AMA could increase patient adherence in patients treated with chemotherapy for chronic lymphocytic leukemia [5].

Based on these findings and considerations, we concluded that AMA was a simple and relatively inexpensive procedure that could be applied to LMIC patients and that had the potential to efficiently reduce refusal or abandonment of therapy or to improve observance in treated patients.

For this reason, we designed a randomized study comparing the standard survey procedure and AMA (here designated as AMAFRICA) in a cohort of 100 lymphoma patients treated with chemotherapy in the Clinical Hematology Department of Abidjan University Medical Center (Ivory Coast). This study was in part supported by Pierre Fabre Foundation, a not-for-profit charitable organization involved in health care in French-speaking LMICs, especially in South Asia and Africa.

## Methods

### Patient eligibility

All patients referred to our center (Yopougon University Medical Center, Abidjan) with newly diagnosed Hodgkin or non-Hodgkin lymphoma (HL or NHL), or endemic Burkitt lymphoma and aged 5 to 75 years, were eligible to participate in the AMAFRICA study. Recruitment was based on convenience sampling. Eligibility was based on provisional diagnosis of lymphoma as established by local pathologist (quoted as “referral pathologist” in opposition to the “expert”, see below).

### Patient characteristics

In this study, we prospectively collected information relative to gender, age, marital status (living with a partner versus living alone), employment, residency (Abidjan city versus others), income (< 100 USD versus > 100 USD per month), and comorbidity (Charlson’s score).

### Diagnosis

Diagnosis of ML was based either on biopsy specimens or on cytological and immunological analyses in the presence of circulating malignant cells. Immunophenotype analysis of peripheral mononuclear circulating cells were performed in Cerba® Laboratory (a private institution located in Paris, France) and resulted in the characterization of B- or T-derived main subtypes (CD3, CD8, CD4, CD8, CD56, CD19, CD20). Biopsy specimens were processed at the Pathology Department of Treichville University Medical Center in Abidjan. At this level, morphological examination was performed after HE staining without immunohistochemistry (IHC).

However, materials were also addressed in duplicate to the Pathology Department of the Toulouse University Medical Center (France), Prof. Camille Laurent as referent. The reviewing procedure was performed not only with morphology analysis, but also with IHC using a combination of monoclonal antibodies and more occasionally molecular biology according to standardized procedures [6]. Patients with insufficient materials resulting in uncertainty about ML diagnosis were excluded. According to the centralized histopathological review we used in France for lymphoma diagnosis (the Lymphopath Procedure), discordance between referral and expert referred to any change made by the expert on the basis of the WHO lymphoma classification [6].

### Clinical management

At diagnosis, routine biological analyses (blood cell count, LDH, hepatic enzymes, CRP) as well as a CT Scan were performed for each patient. Post-treatment routine biological analysis and CT scan were performed only for patients who achieved their therapeutic plan.

The chemotherapy was planned as follows: For HL: ABVD protocol for 6 cycles. For NHL, CHOP protocol 6 cycles or RCHOP (CHOP 6 cycles + Rituximab 375 mg/m^2^ at each cycle). Rituximab was used for patients with insurance (*n* = 10). Burkitt lymphomas (BL) were treated with the CMA regimen as described previously by one of us [7]. Importantly, costs related to transportation, hospitalization and drugs remained payable to patients.

### The AMAFRICA procedure

AMAFRICA started when the NN met the patient for the first time and assisted the oncologist in informing the patient about the objectives of the study and the methods, including the randomization (AMAFRICA versus standard). In case of acceptance, informed consent was collected and biopsy specimens were sent to France for review. Randomization was realized in France, based on provisional diagnosis (before the review). However, only patients with confirmed diagnosis of lymphoma (after review) entered into the study. The expert review was done in a timely fashion, so chemotherapy was administered within a reasonable time limit.

Once included, patients assigned to the AMAFRICA group received free cellular phones to communicate with the NN during treatment.

During therapy, according to the AMA procedure previously described [4], the NN called the patients weekly and collected all information related to treatment-induced toxicity (notably, fever, respiratory signs, mucositis, pains, digestive troubles). These data were recorded in a clinical report form which was addressed to the oncologist. The NN was trained for a period of one month in France.

The study was started in May 2015 and recruitment ended in November 2017 (*n* = 100 patients included).

### Costs

Costs related to transportation, hospitalization and drugs were payable to patients. Pierre Fabre Foundation covered the costs related to the study itself including: Nurse Navigator salary, transportation and analysis of biopsy specimens, CT scan and routine biology for staging and post-treatment evaluation. Pathological review in Toulouse was free, including reagents and working time.

### Ethics and consent to participate

The study was approved by the Ethical Committee of Abidjan University Medical Center (N°027/MSLS/CNER-dkn). The informed consent obtained from study participants was written.

### Judgment criteria

Refusal referred to patients who entered into the study but refused chemotherapy.

Abandonment referred to patients who entered into the study, received at least 1 cycle and then decided to stop therapy.

Non-adherent patients referred to patients who entered into the study, achieved their chemotherapy plans, but received less than 75% of the theoretical total dose for doxorubicin and/or cyclophosphamide.

Adherent patients referred to patients who entered into the study, achieved their chemotherapy plans, and received at least 75% of the theoretical total dose for doxorubicin and cyclophosphamide.

Complete response was based on intent-to-treat and assessed according to Cheson’s criteria 1999 [8].

Overall survival was measured from the entry into the study; any cause of death was considered.

### Data collections and analysis

SPSS (IBM Corporation, Armonk, NY, USA) was used to conduct data management and analysis. For each variable, frequency distributions, median, means, and standard deviations were calculated. Differences in socio-demographic and clinical characteristics between the two groups were compared using chi-square, Fisher’s exact, and t-tests. Fisher’s exact test for variables with over two categories was executed in R version 2.15.0. Survival analysis was performed using the Kaplan-Meier method. Overall survival was calculated from treatment initiation to death from any cause or at the date of the last visit. For comparison of cohorts, the Mann-Whitney test was employed.

## Results

### Patients

100 patients entered into the study. Socio-demographics are listed in Table 1. Most patients were young adults without comorbidity, employed and lived with a partner. Thirteen per cent of patients were between 10 and 30 years old. Near 60% lived in Abidjan or environs, while about one third lived outside and sometimes very far away (400–600 km). Patient income was below 127 USD per month for all patients (mean for Africa is about 156 USD) and 77% reported even less (below 100 USD). Socio-demographic patterns were similar between AMA and non-AMA groups (Table 1).

Diagnosis: for 15% of cases, diagnosis was established through examination of circulating malignant cells by combining morphology and immunophenotype analyses. In other cases, diagnosis was established through biopsy specimen. For the latter, a provisional diagnosis was proposed by the local pathologist referral before materials were examined by the expert. The final diagnoses established after expert review are depicted in Table 2. The discordance rate between referral (based on HE) and expert (based on IHC) was 58.8%. Discordances are described in details in the foot note section of Table 3. With regards to potential therapeutic implications, the most concerning discordances were for MCL, MZL, T-ALL/LL, ALCL while discordance rates were much lower for HL, follicular lymphoma (or Burkitt Lymphoma. The distribution of diagnoses reflected recruitment based on convenience sampling. For example, Burkitt lymphomas were underrepresented in our cohort because of the creation of a pediatric oncology unit in another city of Ivory Coast. The most frequent ML subtype was DLBCL (25 cases). No differences were found between AMA and non-AMA groups (see Table 2).

### Feasibility

in the AMAFRICA group, the NN performed a total of 364 phone calls, among which 72 were missed calls (19.8%). Missed calls were more frequent among patients living outside Abidjan, older than 50 years, and with poor income (data not shown). For the remaining patients, they were punctual in answering scheduled calls and grateful to the nurse navigator. The procedure was uniformly appreciated by patients, informal caregivers and medical staff.

### Impact of the AMAFRICA procedure

For the entire cohort, refusal and abandonment rates were as high as 43 and 17%, respectively. Refusal and abandonment were observed equally for DLBCL, HL or T cell lymphoma. However, the AMA group displayed significantly lower rates of refusal and abandonment, compared to controls (see Table 4). Furthermore, only 29 patients completed therapy, among whom 9 (31%) were treated with more than 25% reduction of dose intensity (non-adherent). Finally, only 20 patients received full doses of chemotherapy (adherent). Complete response (CR) rate being calculated as intent-to-treat is low and similar in the two groups (about 16%).

### Reasons for refusal

as a secondary objective, we asked patients the main reason for which they decided to not be treated. We found: personal decision in relation to lack of financial support (46%), family opposition (which can include financial reasons) (15%), interference with traditional medicine (11%), transportation obstacles (7%) discouragement (7%) or various other reasons (14%).

### Survival

**Fig. 1:**
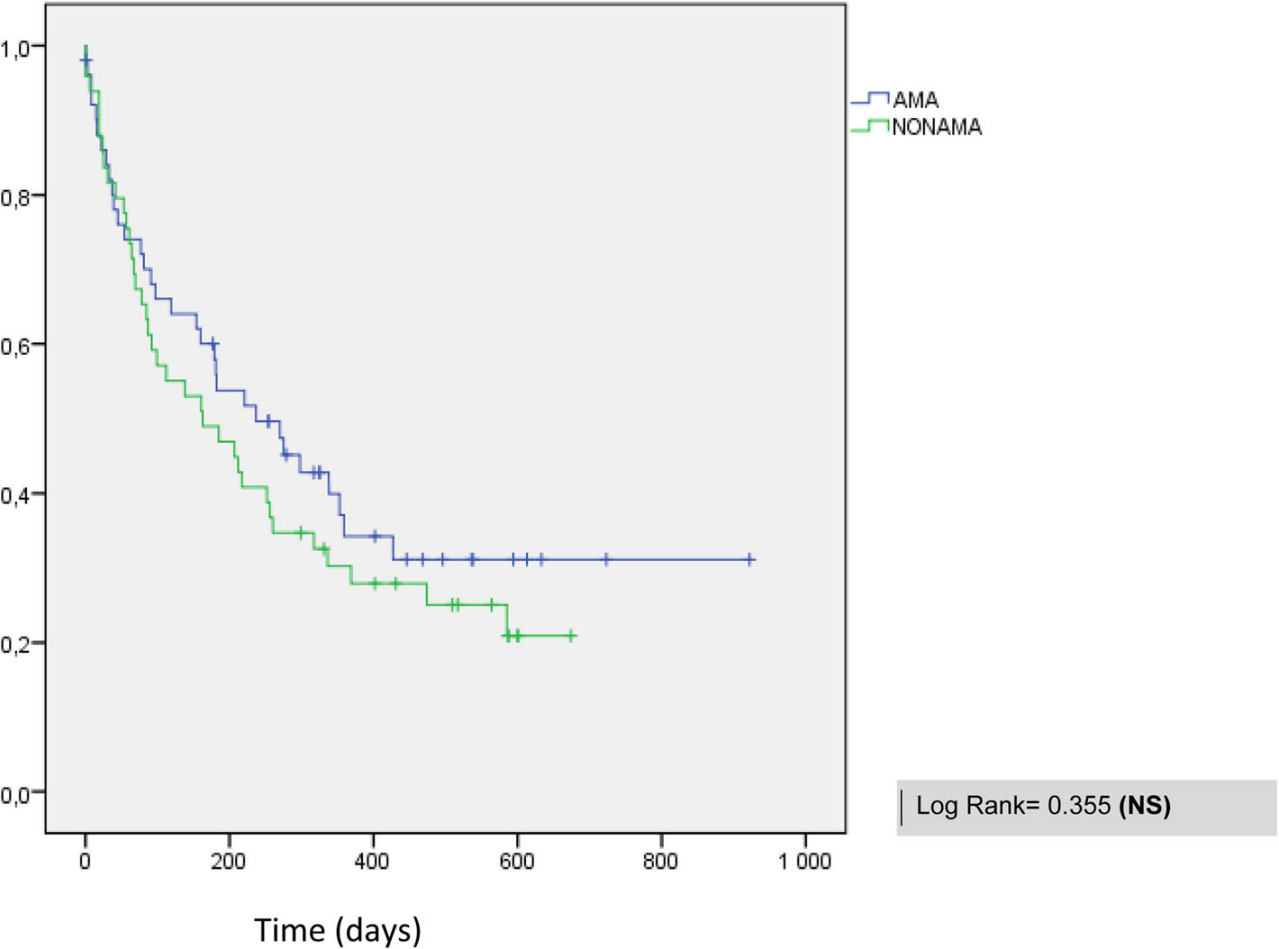
Overall survival in AMAFRICA and non-AMAFRICA groups

overall survival (OS) was calculated from the entry into the study. Median global survival was only 6 months for the entire cohort. However, when applied to patients who achieved full dose treatment (20%), results were much better with CR rate of about 50% and median OS above one year (data not shown). No differences were detected between the two groups (Fig. 1).

**Table 1:** Socio-demographics of patients

Parameters	AMAFRICA *n* = 51	Standard *n* = 49	*P* value
Gender, n (%)	M = 30 (30%) F = 21 (31%)	M = 30 (30%) F = 19 (19%)	*P* = 0.80
Age, years median (min-max.)	51 (10–78)	49 (9–74)	*P* = 0.31
Marital status (*n* = 98), n (%)			
Living with partner	40 (41%)	41 (42%)	*P* = 0.669
Living alone	9 (9%)	8 (8%)	
Employment n (%)			
Employed (yes)	48 (48%)	45 (45%)	*P* = 0.953
Unemployed (no)	3 (3%)	4 (5%)	
Residency (n = 98), n (%)			
Urban	29 (30%)	32 (33%)	*P* = 0.957
Rural	20 (20%)	17 (17)	
Income, n (%)			
Low (< 100 USD per month)	38 (38%)	39 (39%)	*P* = 0.706
Middle(127–100 USD per month	13 (13%)	10 (10%)	
Comorbidity (Charlson score), n (%)			
0	40 (40%)	42 (42%)	*P* = 0.239
> 0	11 (11%)	7 (7%)

**Table 2:** a. Lymphoma subtypes

Histological subtypes (expert diagnosis)	AMA group	Non-AMA group	total
DLBCL	13	12	25
BURKITT	4	0	4
FL	4	4	8
MCL	1	4	5
CLL/PLL	5	7	12
MZL	8	9	17
PTCL	4	4	8
T-ALL/LL	3	1	4
ALCL	1	2	3
HL	8	6	14
Total	51	49	100

Abbreviations: *ALCL* (anaplastic large cell lymphoma); *CLL* (chronic lymphocytic leukemia); *DLBCL* (diffuse large B cell lymphoma); *FL*: (Follicular Lymphoma); *HL* (Hodgkin Lymphoma); *MCL* (Mantle cell lymphoma); *MZL* (Marginal Zone Lymphoma); *NHL* (Non- Hodgkin Lymphoma); *PLL* (prolymphocytic leukemia); *T-ALL/LL* (T-acute lymphoblastic leukemia/lymphoblastic lymphoma)

**Table 3:** b. Discrepancies between referral and expert pathologists based on tissue section analysis

Nature of the discrepancy*	Number of cases
Low grade B cell lymphoma to diffuse large B cell lymphoma [1]	15
Unclassified T cell proliferation to classified lymphoma subtypes [2]	11
Unclassified lymphoma to classified lymphoma subtype [3]	10
Low grade B cell lymphoma reclassification [4]	8
NHL to HL [5]	3
Reactive lesion to NHL [6]	3
Total	50 /85** (58.8%)

*from provisional to expert diagnosis

**based on 85 biopsy specimens, the 15 remaining cases were characterized on the basis of circulating malignant cells analysis (morphology and immunophenotype)

Details of expert review [1]: 15 cases referred as FL (n = 5), CLL (*n* = 3), Burkitt lymphoma (*n* = 3) and PTCL (n = 4) were changed to DLBCL NOS [2]. 11 cases referred as unclassified T-cell lymphoproliferation to classified lymphoma subtype: ALCL (*n* = 2), PTCL (*n* = 1), T-ALL/LL (n = 4), HL (n = 3) and DLBCL NOS (n = 1) [3]. 10 unclassified lymphomas were classified in PTCL (n = 1); MZL (*n* = 6), MCL (n = 2), CLL (n = 1) [4]. This category includes MZL (n = 6) and MCL (n = 2) [5]. This category includes 3 NHL (3 DLBCL) that were changed to HL (n = 3) [6]; 3 reactive lesions were changed to HL (n = 2) and FL (n = 1)

**Table 4:** Impact of the AMAFRICA procedure on treatment

	AMAFRICA (n = 51)	Non-AMAFRICA (n = 49)	Entire cohort
Refusal	17	26	*p* = 0.047
- Discouragement	1	2	
- Transport obstacles	2	1	
- Financial reasons	6	14	
- Familly opposition	2	3	
- Traditional medicine	3	3	
- Others	3	3	
Abandonment	5	12	*p* = 0.046
- Discouragement-Disappearance of tumor syndrome	01	30	
- Financial reasons	0	6	
- Transport obstacles	1	1	
- Traditional medicine	0	1	
- Others	3	1	
Treatment completed	16	13	*p* = 0.59
Complete response (%)	15.6%	16.3%	*p* = 0.93

## Discussion

This study investigated the impact of AMAFRICA procedure, a patient navigator patient program, on the management of patients treated for ML in Ivory Coast. This randomized study showed a significant impact of AMAFRICA with the rate of refusal and abandonment. However, response rate and overall survival were unaffected.

AMAFRICA was derived from AMA, a patient navigator variant used in France for the management of chemotherapy intercourse for ML patients. According to this procedure, a specialized nurse (“nurse navigator”/NN) performed a systematic outgoing phone call at the patient’s home, collected all information and served as coordinator between the oncology unit and the patient [4]. AMA is now used as a standard in numerous French institutions, including ours. Nearly 3000 patients have been enrolled. More recently, AMA was used for monitoring lymphoma patient survivors [9]. Previous studies have suggested that the AMA procedure could improve the quality of clinical management, including observance, safety, comfort and reassurance [4,5]. We reasoned that such a procedure could be useful in LMIC to prevent refusal or abandonment of therapy, two major limitations of cancer care in underserved countries.

Ivory Coast is one of the West African French-speaking countries with 25 million inhabitants, with nearly one third living in Abidjan, the capital city. Ivory Coast has a gross domestic product (GDP) of about 1662 USD per capita (3130 PPP/Purchasing Power Parity) and a human development index (HDI) of 0.474 (182nd position in the world). Despite a fast-growing economy, Ivory Coast meets health financing criteria of low-income countries by limitation of universal health care and large contributions of out-of-pocket financing [10]. Thus, whereas total health spending per capita was PPP 172 USD, 51% of this amount was paid out-of-pocket by households, (www.africanstrategies4health.org) and less than 10% of the Ivorian population has adequate health coverage. Despite these limitations, health care demand remains high. For example, in 2018, the hematology department of Abidjan University Medical center managed 1142 patients, among them 159 cases of ML.

The rate of treatment refusal and abandonment is very high (60%). Similar rates of treatment refusal have been also described in other countries such as India [11] and Kenya [12]. In a meta-analysis, Gupta and coworkers made an inventory of 83 studies in various LMIC (mainly in Asia), and found a global refusal/abandonment rate of 54% [13]. However, most of these studies were retrospective. We believe that the rate we found is more accurate to reflect health care access in the real African world. This rate is largely explained by financial constraints.

In this context, encouraging patients to be treated is challenging. Indeed, a large majority of patients who abandoned (a total of 17%), left after only one cycle, again mostly for financial reasons. The cost of each CHOP cycle has been estimated at 376 USD. Thus, the cost of complete CHOP therapy represents about 70% of the mean annual PPP income, while spending more than 10% of total expenditure on out-of-pocket health care costs is considered by WHO as a threshold of so-called “catastrophic health spending” [10]. Despite some occasional support (including family generosity), this situation poses a threat in terms of dispossession and misery. Therefore, any plan of treatment should be considered cautiously, based on patient financial resources.

Financial considerations are the main reason put forward by the patients to justify refusal and abandonment. Family opposition is often also based on costs. However, our study revealed other reasons such as transportation, already documented in other LMIC [14], or cultural reasons such as fatalism or interference with traditional medicine, also documented elsewhere [12]. All these factors also play a role in delay of clinical management and the high incidence of advanced forms of diseases.

Reports dealing with treatment efficacy in LMIC cancer patients based on intent-to-treat are uncommon. Our study shows poor response rate and survival based on intent-to-treat in lymphoma patients, compared to developed countries. For example, the CHOP regimen yielded about 50% CR rate and RCHOP (the standard in Europe and in North America) about 70% or even more, depending on the initial risk factors. These results should be compared with the 16% CR rate of our cohort. The combination of delayed diagnosis, treatment refusal or abandonment, poor adherence to treatment and death under therapy converge to yield a low rate of response, even if adherent patients displayed an encouraging 50% CR rate. Another implication of the high rate of refusal and abandonment we described herein is that future prospective clinical trials should take into account such limitation for calculating the number of patients needed to be recruited in order to avoid not conclusive study.

The North/South cooperation built for this study was also instructive for pathological review. Indeed, the provisional diagnosis provided by the local pathologist was changed in about 60% after reanalysis by an expert using IHC. Such high rate of discordance could be judged as alarming. Since the referral (here our pathologist colleague working in Abidjan) used HE staining without IHC, a high discordance rate was anticipated, especially for classifying small B-cell and T cell lymphoma. As a comparison, the rate of discordance in France between referral pathologist and expert was 20%, nearly half of cases corresponding to B-cell derived NHL subtype reclassification [6]. there is no doubt that pathological analysis should be improved in Ivory Coast. In our study, specimen reanalysis was found to be feasible for a reasonable cost (about 90 USD for air transportation). However, this process is not satisfactory. In the near future, IHC should be implemented in Abidjan and in anticipation, one of our Ivorian pathologist colleagues has received specific training in IHC procedure and interpretation. Telepathology represents also an attractive approach [15].

## Conclusion

Finally, this prospective study confirms that treatment refusal and abandonment are major issues for treatment of ML in LMIC. Some parameters should be considered such as education, communication with patients and families, access to medical staff, motivation and logistics. However, the main obstacles are financial. It is very likely that the limitation of universal health care plays a role even if it is still debated [16]. Our study may have implications for health care policy applied to ML management. Indeed, the financing of “essential medicine” [17] like CHOP for the whole population appeared more critical that the introduction of new drugs, such as targeted therapies, including under their generic forms. At the least, further studies are needed to measure cost efficacy studies, for example through DALY calculation [18]. This approach has been already developed in Brazil and Malawi in the context of pediatric malignancies [19]. Finally, since they are feasible, PN-derived procedures like AMA could improve patient management in other less aggressive clinical settings such as in infectious or degenerative chronic diseases, as well as in sickle cell anemia.

## Data Availability

The datasets used and/or analysed during the current study are available from the corresponding author on reasonable request.

## Abbreviations

ABVD: Adriamycin bleomycin vinblastine dacarbazine
AMA: Assistance des malades ambulatoires
CHOP: Cyclophosphamide hydoxydaunorubicin oncovin prednisolone
CLL: Chronic lymphocytic leukemia
CR: Complete response rate
DLBCL: Diffuse Large B Cell Lymphoma
FL: Follicular Lymphoma
HL: Hodgkin Lymphoma
IHC: Immunohistochemistry
LMIC: Low-middle income countries
MCL: Mantle cell lymphoma
ML: malignant lymphoma
MZL: Marginal Zone Lymphoma
NHL: Non- Hodgkin Lymphoma
NN: Nurse navigator
OS: Overall survival
PLL: Prolymphocytic leukemia
PN: Patient Navigator
RCHOP: Rituximab cyclophosphamide hydoxydaunorubicin oncovin prednisolone
US: United States
